# Mathematical modeling of the effects of glutathione on arsenic methylation

**DOI:** 10.1186/1742-4682-11-20

**Published:** 2014-05-16

**Authors:** Sean D Lawley, Jina Yun, Mary V Gamble, Megan N Hall, Michael C Reed, H Frederik Nijhout

**Affiliations:** 1 Department of Mathematics, Duke University, Durham, NC 27708, USA; 2 Department of Environmental Health Sciences, Mailman School of Public Health, Columbia University, New York, USA; 3 Department of Epidemiology, Mailman School of Public Health, Columbia University, New York, USA; 4 Department of Biology, Duke University, Durham, NC 27708, USA

**Keywords:** Mathematical model, Arsenic, Methylation, Glutathione, Detoxification

## Abstract

**Background:**

Arsenic is a major environmental toxin that is detoxified in the liver by biochemical mechanisms that are still under study. In the traditional metabolic pathway, arsenic undergoes two methylation reactions, each followed by a reduction, after which it is exported and released in the urine. Recent experiments show that glutathione plays an important role in arsenic detoxification and an alternative biochemical pathway has been proposed in which arsenic is first conjugated by glutathione after which the conjugates are methylated. In addition, in rats arsenic-glutathione conjugates can be exported into the plasma and removed by the liver in the bile.

**Methods:**

We have developed a mathematical model for arsenic biochemistry that includes three mechanisms by which glutathione affects arsenic methylation: glutathione increases the speed of the reduction steps; glutathione affects the activity of arsenic methyltranferase; glutathione sequesters inorganic arsenic and its methylated downstream products. The model is based as much as possible on the known biochemistry of arsenic methylation derived from cellular and experimental studies.

**Results:**

We show that the model predicts and helps explain recent experimental data on the effects of glutathione on arsenic methylation. We explain why the experimental data imply that monomethyl arsonic acid inhibits the second methylation step. The model predicts time course data from recent experimental studies. We explain why increasing glutathione when it is low increases arsenic methylation and that at very high concentrations increasing glutathione decreases methylation. We explain why the possible temporal variation of the glutathione concentration affects the interpretation of experimental studies that last hours.

**Conclusions:**

The mathematical model aids in the interpretation of data from recent experimental studies and shows that the Challenger pathway of arsenic methylation, supplemented by the glutathione effects described above, is sufficient to understand and predict recent experimental data. More experimental studies are needed to explicate the detailed mechanisms of action of glutathione on arsenic methylation. Recent experimental work on the effects of glutathione on arsenic methylation and our modeling study suggest that supplements that increase hepatic glutathione production should be considered as strategies to reduce adverse health effects in affected populations.

## Introduction

Arsenic is a naturally occuring metalloid that finds its way into the food chain through water, plants, and animals. In many parts of the world, arsenic is a major health hazard [1-3]. Chronic arsenic exposure has been associated with cancer, heart disease, neuropathies, and with deficits in intelligence in children [4,5]. Arsenic is mainly ingested as inorganic arsenic, iAs. The metabolism of arsenic in the liver has traditionally been thought to proceed via successive enzymatic methylations to methylarsonic acid, MMAs ^V^, and dimethylarsinic acid DMAs ^V^, with two intervening reduction steps [6-8]. This is known as the Challenger pathway and the methylations are catalyzed by arsenic methyltransferase, AS3MT. The Challenger pathway has been considered a detoxification pathway because reactive oxygens are replaced by methyl groups and DMAs ^V^ is readily exported from the liver and excreted in urine. However, there is considerable evidence that the intermediate trivalent MMAs is equally or more toxic than inorganic arsenic or DMAs ^V^[9-11].

In recent years, evidence has been accumulating that the tripeptide glutathione, GSH, plays an important role in the Challenger pathway. Since GSH is a reductant, it increases the rates of the reduction steps [12-14] and glutathione S-transferase has been shown to help convert MMAs ^V^ to MMAs ^III^ in different tissues [15,16]. Thomas, Styblo and colleagues [17-19] have studied methylation in the presence of other reductants as well as GSH. Even in the presence of other reductants, GSH increases methylation yield, and Song et al. [20] suggested that GSH increases the activity of AS3MT. In addition, in the experiments of both [20] and [19], it is shown that increasing GSH concentration when the concentration is low increases methylation rate, but increasing GSH concentration when GSH concentration is high decreases methylation rate. Finally, Hayakawa et al. [21] have proposed an alternate pathway for methylation in which only the arsenicals bound to GSH can be methylated.

**Figure 1:**
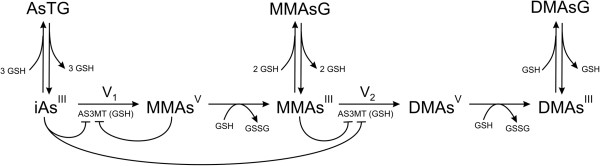
**The reaction diagram. **The diagram depicts the traditional Challenger pathway [6] augmented by three effects of glutathione. *V *_1_and *V *_2_are the velocities of the methylation steps. GSH and other reductants increase the velocity of the reduction reactions. GSH increases the activation of AS3MT. GSH conjugates and sequesters the arsenicals iAs, MMAs ^III^, and DMAs ^III^. Although not indicated, the half-life of GSH in reaction mixtures is taken into account. The various inhibitions of iAs and MMAs ^III ^are indicated. The kinetics of *V*_1 _and *V*_2 _and the functional form, *U *(*GSH*) by which GSH affects AS3MT are given in the Methods.

In a complicated physiological and biochemical situation such as this, mathematical modeling can be a useful tool for sorting out the consequences of different hypotheses and for helping to interpret experimental data. We have made a mathematical model of the arsenic methylation pathway which incorporates three different roles for GSH. First, GSH, as well as other reductants, drives the reduction from valence 5 to valence 3 arsenicals. Second, GSH activates AS3MT. Third, GSH binds reversibly to and sequesters all three arsenic species, iAs, MMAs ^III^, and DMAs ^III^. In our model, the GSH-conjugated arsenicals are not further methylated. We include in the model the many known inhibitions of the methylation reactions and include a new inhibition suggested by the data in [19]. The model is depicted in Figure 1; details are given in Methods. We use the model to explain and interpret the experimental data in [20] and [19]. In particular, we show that the experimental data can be explained by the traditional Challenger pathway with the GSH effects outlined above, so it is not necessary to assume that only GSH-conjugated arsenicals can by methylated as proposed by Hayakawa [21].

Cullen [22] discusses the current state of knowledge of methylation of arsenic and outlines four different detailed mechanisms. Considerable knowledge is now available on which cysteine residues in AS3MT are necessary for methylation and on the order of the reaction steps [23,24]. And, it is known [19] that other thiols besides GSH affect methylation and that there may be an interaction between these thiols and GSH. On the physiological level, GSH is in high concentration in cells and can effect transport processes that control arsenic uptake and removal from cells, as well as the availability of other thiols. Furthermore, GSH is known to bind to xenobiotics, including metals, and, indeed, arsenic-glutathione conjugates appear in the bile of rats fed arsenic containing diets [25], so arsenic conjugation may be an important arsenic excretion pathway. None of these details is in our model.

There are different kinds of experiments, and corresponding models, that shed light on arsenic methylation and arsenic detoxification. There are studies in humans where arsenic metabolites are measured in urine and blood [8,26-29]. There are cell culture experiments in which arsenicals are typically measured in the external medium [10]. And, there are experiments in which reaction mixtures of arsenicals, AS3MT, and various other metabolites are prepared [17-21]. A number of pharmacokinetic models have been used to interpret data in these different experimental situations. We have previously constructed a whole body model of arsenic methylation [30] and compared the results to the clinical results of Buchet et al. [26,27] and the clinical trial of Gamble et al. in Bangladesh [28,29]. There are other whole body models [31-35]. We used a reduced version of our whole body model to study the cell culture experiments in [36]. Previous models for these cell culture experiments were created in [37,38]. In two recent papers, Georgopoulos and coworkers create mathematical models based on the Hayakawa pathway to study hepatocyte culture experiments including GSH conjugation, reactive oxygen species, and DNA damage [39,40].

Our model, which investigates the three “effects” of GSH described above and depicted in Figure 1, builds on our previous model of arsenic detoxicfication [30]. Although the model simplifies complicated and interesting biochemical and physiological questions that are the object of current investigations, it enables us to understand three important effects of GSH on arsenic methylation. It is vital to understand the effects of GSH on detoxification mechanisms in hepatocytes, because such understanding may give important information on whether substrates like N-acetyl-cysteine that increase liver GSH may be useful supplements in regions of the world where arsenic is endemic in the water or food supply.

## Methods

A diagram of the reactions in our model is depicted in Figure 1. The variables in the model are defined in Table 1, followed by the differential equations and Table 2, which gives the values of the rate constants. After Table 2, the functions *U*,*V*_1_,*V*_2_ in the differential equations are defined and important modeling issues are discussed.

$$\begin{array}{ll} \frac{d[\;\text{iAs}]}{\text{dt}} & =U(\text{[GSH]})\cdot V_{1}([\;\text{iAs}]\;,[\;{\text{MMAs}}^{\text{V}}]\;)-k_{1}{\left[\;\text{GSH}\right]}^{3}[\;\text{iAs}]+\;k_{-1}[\;\text{ATG}]\\ \frac{d[\;{\text{MMAs}}^{\text{V}}]}{\text{dt}} & =k_{6}U(\text{GSH})\cdot V_{1}([\;\text{iAs}]\;,[\;{\text{MMAs}}^{\text{V}}]\;)-(k_{5}+k_{6}[\;\text{GSH}]\;)[\;{\text{MMAs}}^{\text{V}}]\\ \frac{d[\;{\text{MMAs}}^{\text{III}}]}{\text{dt}} & =(k_{5}+k_{6}[\;\text{GSH}]\;)[\;{\text{MMAs}}^{\text{V}}]-\;U(\text{[GSH]})\cdot V_{2}([\;{\text{MMAs}}^{\text{III}}]\;,[\;\text{iAs}]\;)\\ & \;-k_{2}[\;{\text{MMAs}}^{\text{III}}]{\left[\text{GSH}\right]}^{2}+\;k_{-2}[\text{MMAsG}]\\ \frac{d[\;{\text{DMAs}}^{\text{V}}]}{\text{dt}} & =U(\text{GSH})\cdot V_{2}([\;{\text{MMAs}}^{\text{III}}]\;,[\text{iAs}]\;)-(k_{7}+k_{8}[\;\text{GSH}]\;)[\;{\text{DMAs}}^{\text{V}}]\\ \frac{d[{\text{DMAs}}^{\text{III}}]}{\text{dt}} & =(k_{7}+k_{8}[\;\text{GSH}]\;)[{\text{DMAs}}^{\text{V}}]-\;k_{3}[\;{\text{DMAs}}^{\text{III}}][\;\text{GSH}]+\;k_{-3}[\;\text{DMAsG}]\\ \frac{d[\;\text{ATG}]}{\text{dt}} & =k_{1}{\left[\;\text{GSH}\right]}^{3}[\;\text{iAs}]-\;k_{-1}[\;\text{ATG}]\\ \frac{d[\;\text{GSH}]}{\text{dt}} & =-\;k_{4}[\;\text{GSH}]-\;3k_{1}{\left[\;\text{GSH}\right]}^{3}[\;\text{iAs}]+\;3k_{-1}[\;\text{ATG}]-\;2k_{2}{\left[\;\text{GSH}\right]}^{2}[\;{\text{MMAs}}^{\text{III}}]\\ & \;+2k_{-2}[\;\text{MMAG}]-\;k_{3}[\;\text{GSH}][\;{\text{DMAs}}^{\text{III}}]+\;k_{-3}[\;\text{DMAG}]\\ \frac{d[\;\text{MMAG}]}{\text{dt}} & =k_{2}{\left[\;\text{GSH}\right]}^{2}[\;{\text{MMAs}}^{\text{III}}]-\;k_{-2}[\;\text{MMAG}]\\ \frac{d[\;\text{DMAG}]}{\text{dt}} & =k_{3}[\;\text{GSH}][\;{\text{DMAs}}^{\text{III}}]+\;k_{-3}[\;\text{DMAG}] \end{array}$$

**Table 1:** **Variables in the model ( *μ *** **M)**

	
iAs	Inorganic arsenic
MMAs^III^	Monomethylarsonous acid
MMAs^V^	Monomethylarsonic acid
DMAs^III^	Dimethylarsinous acid
DMAs^V^	Dimethylarsinic acid
AsTG	Arsenic triglutathione
GSH	Glutathione
MMAsG	Monomethylarsenic diglutathione
DMAsG	Dimethylarsenic glutathione

**Table 2:** **Rate constants in the model ( *μ *** **M/hr)**

		
*k*_1 _= 10^-11^	*k*_-1 _= 375	iAs ⇆AsTG
*k*_2 _= 10^-5^	*k*_-2 _= .25	MMAs ⇆MAsDG
*k*_3 _= 10^-3^	*k*_-3 _= 10^-3^	DMAs ⇆DAsG
*k*_4 _= ln(2)/2.5		GSH decay
*k*_5 _= 100	*k*_6 _= .1	Reduction of MMAs^V^
*k*_7 _= 5	*k*_8 _=.1	Reduction of DMAs^V^
*V*_1_	*K*_*m *_= 4.6	*K*_*m *_for [iAs]
	$$K_{i}^{A}=1.26$$	
	$$K_{i}^{M}=40$$	
*V*_2_	*K*_*m *_= 4.6	*K*_*m *_for [iAs]
	$$K_{i}^{A2}=40$$	
	$$K_{i}^{M2}=\sqrt{6}$$	

### Methylation reactions

The velocity, *V*_1_, of the reaction in which iAs^III^ is methylated to become MMAs^V^ is given by:

$$V_{1}([\;{\text{iAs}}^{\text{III}}]\;,[\;{\text{MMAs}}^{\text{V}}]\;)\;=\;\frac{V_{\text{max}}[\;{\text{iAs}}^{\text{III}}]}{\left(K_{m}\;+\;[\;{\text{iAs}}^{\text{III}}]\right)\left(1+\frac{\left[{\text{iAs}}^{\text{III}}\right]}{K_{i}^{A}}\right)\left(1+\frac{\left[{\text{MMAs}}^{\text{V}}\right]}{K_{i}^{M}}\right)}.$$

We use the value *K*_*m *_= 4.6 *μ *M for the Michaelis-Menten constant for AS3MT for iAs^III^ as found in [41]. The reaction has substrate inhibition by iAs^III^; we take the inhibition constant to be $K_{i}^{A}=1.26\;\mu$M as found in [42]. It is known that this reaction is inhibited by the product MMAs^V^ and we take the inhibition constant, $K_{i}^{M}=40\;\mu$M from [35] and [43]. We note that it is not certain that the enzyme investigated in [43] is identical to AS3MT.

The velocity, *V*_2_, of the reaction in which MMAs^III^ is methylated to become DMAs^V^ is given by:

$$V_{2}([\;{\text{MMAs}}^{\text{III}}]\;,[\;{\text{iAs}}^{\text{III}}]\;)\;=\;\frac{V_{\text{max}}[\;{\text{MMAs}}^{\text{III}}]}{\left(K_{m}\;+\;[\;{\text{MMAs}}^{\text{III}}]\right)\left(1+\frac{\left[{\text{iAs}}^{\text{III}}\right]}{K_{i}^{A2}}\right)\left(1+\frac{{\left[{\text{MMAs}}^{\text{III}}\right]}^{2}}{{\left(K_{i}^{M2}\right)}^{2}}\right)}.$$

As above we take *K*_*m *_= 4.6 *μ *M and we set $K_{i}^{A2}=40\;\mu$M as in [35] and [43]. The inhibition of *V*_2_ by MMAs^III^ is proposed in this paper; the inhibition constant $K_{i}^{M2}=\sqrt{6}\;\mu$M was obtained by fitting the data in [19]. It is reasonable that the second methylation reaction be inhibited by MMAs^III^ since the first methylation reaction is inhibited by MMAs^V^[35,43], though this doesn’t seem to have been remarked on before. We were driven to include this inhibition by the data in [19], their Figure six, which is discussed in detail under Results. The square gave a much better fit of the data, which suggests that the inhibition is cooperative.

### Glutathione as a reductant

It has been known since [12,44,45] that GSH acts to reduce pentavalent to trivalent arsenicals. In cells or *in vivo* other thiols can also act as reductants. We take the rate of the reaction from MMAs^V^ to MMAs^III^ to be *k*_5 _+ *k*_6_[GSH], the *k*_5_ term representing the reduction by other endogenous thiols and the second term representing the reduction by GSH. The concentration of GSH is varied in some of the experiments in [19,20] and in some of our simulations. We take the rate of the reaction from DMAs^V^ to DMAs^III^ to be *k*_7 _+ *k*_8_[GSH] for similar reasons.

### Glutathione affects arsenic methyltransferase

It has been known for a long time that the presence of GSH helps the reduction steps in the methylation chain. The importance of the Styblo data, in [19], Figure six, is that both DMAs^III^ and total DMAs go up by a factor of about four in the presence of GSH. This shows conclusively that GSH increases substantially the activity of AS3MT. We chose a Hill function for the effect of GSH on AS3MT, $U(\text{GSH})=\frac{k_{9}{\left[\text{GSH}\right]}^{5}}{{\left(k_{10}\right)}^{5}+{\left[\text{GSH}\right]}^{5}},$ and the rate constants because they gave a good fit of the data in [19,20].

### Glutathione sequesters arsenic

Arsenic has an affinity for sulfur [12], so it is not surprising that it binds to GSH, especially since a major role of GSH in the liver is to remove xenobiotics including metals. Indeed, arsenic-glutathione compounds can be found in the bile of rats fed arsenic diets [25]. We include in our model the formation of arsenic triglutathione, AsTG, monomethylarsenic diglutathione, MMAsG, and dimethylarsenic glutathione, DMAsG, from iAs ^III^, MMAs ^III^, and DMAs ^III^, respectively. We assume mass-action kinetics and that the reactions are reversible; rate constants are given in Table 2.

## Results

### The Styblo experiments on MMAs^III^

**Figure 2:**
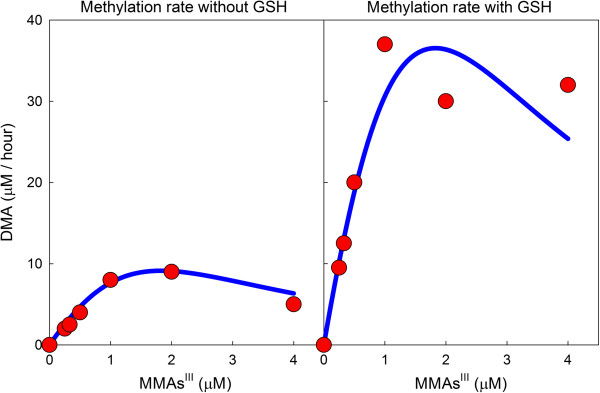
**MMAs **^**III **^**inhibits the second methylation step. **The red dots are data regraphed from Panels C and D (WT) in Figure six of [19]. The blue curves were computed from the mathematical model. For both the data and model curves, the reaction mixture had either 0 mM GSH (left panel) or 1 mM GSH (right panel).

Styblo and colleagues conducted test tube experiments in which MMAs^III^ was methylated to DMAs^V^ and DMAs^III^ in the presence of AS3MT and SAM, both with and without 1 mM GSH [19]. Such experiments on intermediates are particularly valuable for understanding the details of a reaction chain. Both DMAs^III^ and total DMAs were measured and the results are shown in [19], Figure six. Both quantities rise as MMAs^III^ increases when MMAs^III^ is low. However, DMAs^III^ and total DMAs start to decrease as MMAs^III^ gets still larger, showing clear evidence of inhibition of the second methylation step by MMAs^III^. This is reasonable, of course, since MMAs^V^ inhibits the first methylation step, but does not seem to have been commented on before. Our model gives quite good fits to these experiments (see Figure 2), both in the presence and absence of 1 mM GSH.

It has been known for a long time that the presence of GSH helps the reduction steps in the methylation chain. The importance of the data in [19], Figure six is that both DMAs^III^ and total DMAs go up by a factor of about four in the presence of GSH. This shows conclusively that GSH increases substantially the activity of AS3MT.

### Time-course data

**Figure 3:**
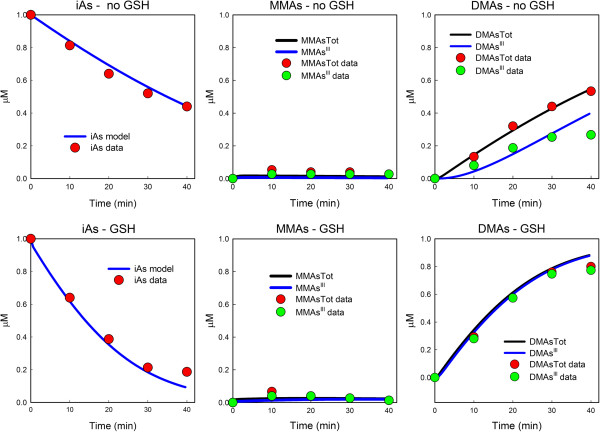
**Time course experiments. ** 1 *μ*M iAs was introduced into a reaction mixture containing 0 mM GSH (upper panels) or 1 mM GSH (lower panels). The red and green dots are data points taken from [19]; the curves are the result of model computations. The experimental measurements did not distinguish between arsenicals and arsenicals bound to GSH, so, likewise, the model curves represent the named arsenicals plus their GSH conjugates. For more detail, see the text.

In one set of experiments in [19], 1 *μ*M of iAs was introduced into a reaction mixture of volume 100 *μ*l that contained 5 *μ*g of recombinant AS3MT, 1 *μ*M of SAM, and either 0 mM or 1 mM GSH. Other reductants were also in the reaction mixture. Over 40 minutes, the concentrations of MMAs^III^, total MMAs, DMAs^III^, and total DMAs were measured. In Figure 3 below, the red and green dots reproduce the data from those experiments originally reported in Figure three, panels A and B in [19] with wild type enzyme. In the left panels of our Figure 3, the data points for iAs were calculated by subtracting total MMAs plus total DMAs from the original amount of iAs, namely 1 *μ*M. We note that the measurements of arsenicals in [19] do not distinguish between arsenicals and arsenicals bound to GSH. The blue and black curves in Figure 3 are model calculations of this experimental situation. The blue curves in the left panels are [iAs] + [AsTG]. In the middle panels, the black curve is [ MMAs^III^] + [ MMAs^V^] + [MMAsG], and the blue curve is [ MMAs^III^] + [MMAsG]. In the right panels, the black curve is [ DMAs^III^] + [ DMAs^V^] + [DMAsG], and the blue curve is [ DMAs^III^] + [DMAsG]. The top row has 0 mM GSH in the reaction mixture and the bottom row has 1 mM GSH in the reaction mixture.

The model curves fit the data points in each panel very well. Note that in the presence of 1 mM GSH more total DMAs is formed and also that there is almost no DMAs^V^ present because, in this experimental context, it is immediately reduced to DMAs^III^, most of which is conjugated with GSH.

### The influence of GSH on methylation

**Figure 4:**
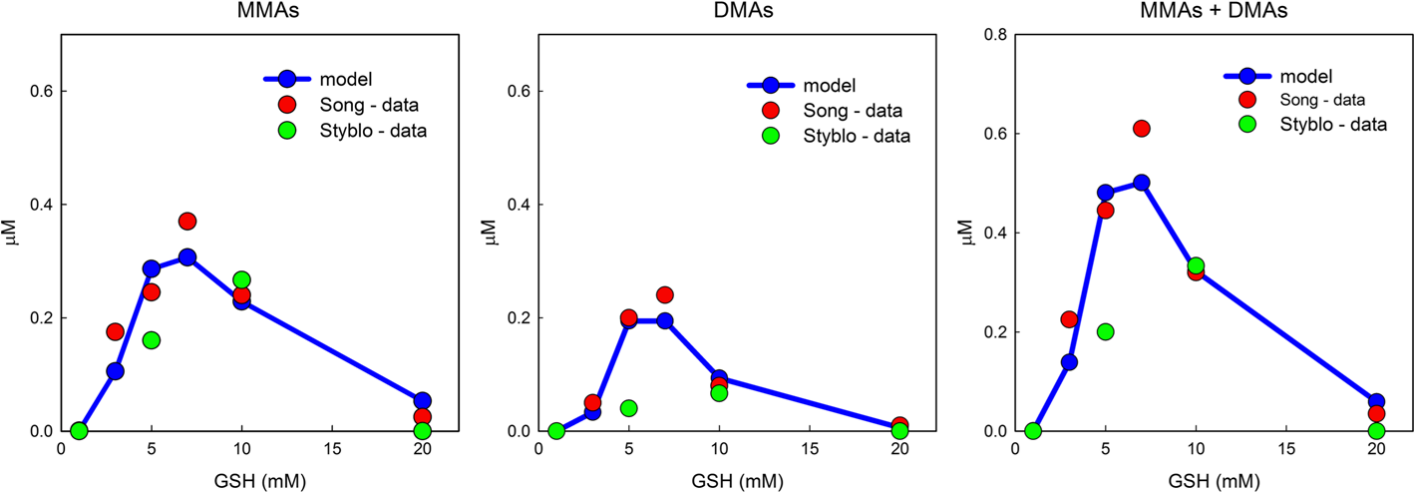
**The influence of GSH on methylation. **1 *μ*M iAs was introduced into reactions mixtures with the indicated amount of GSH. MMAs and DMAs (and their GSH conjugates) were measured after two hours. The green dots and red dots are redrawn data from the experiments in [19], Figure two and [20], Figure six, respectively. The connected blue dots, which are the predictions of our mathematical model, capture the qualitative features of the experimental data.

Both the Styblo group ([19], Figure two) and the Wang group ([20], Figure six) conducted *in vitro* experiments in which different amounts of GSH were incubated in a reaction mixture for two hours and then the amounts of MMAs and DMAs and their GSH conjugates were measured. As reported above, they did not distinguish between the arsenicals and their GSH conjugates. The reaction mixtures were quite similar except that the Wang group had more AS3MT. The Wang group collected data for 1,3,5,7,10,20 mM GSH and the Styblo group for 1,5,10,20 mM GSH. Their results, which are quite similar, are shown as green dots (Styblo) and red dots (Song) in Figure 4. The connected blue dots are the predictions of our model. As one can see, the model predictions capture well the qualitative behavior of both data sets. At low GSH values and at very high GSH values methylation proceeds slowly, but at intermediate values in the range 5–10 *μ*M methylation proceeds much more quickly. Interestingly, this intermediate level is the physiological range of GSH in human hepatocytes [46,47].

### *In silico* experiments

**Figure 5:**
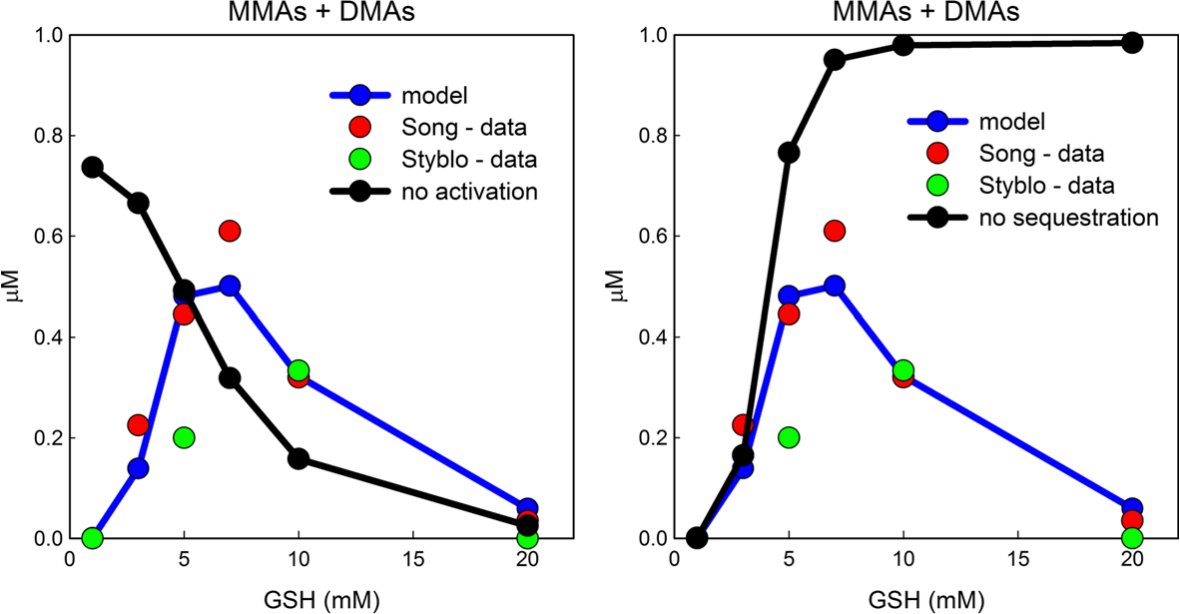
**Removing influences of GSH on methylation. **The left and right panels both reproduce the experimental data, [20] red and [19] green, and blue model curve for MMAs + DMAs with varying amounts of GSH from the right panel of Figure 4. The black curve in the left panel shows what the model fit would be if we removed from the model the excitatory influence of GSH on AS3MT. The black curve in the right panel shows what our model fit would be if we removed the binding of arsenicals to GSH from the model. Clearly, neither black curve fits the data. Both influences of GSH (blue curve) are necessary to explain the experimental data.

One of the advantages of mathematical models is that one can add or remove effects of some variables on other variables to see what difference those effects have. Often it is difficult or impossible to perform the corrresponding biological experiments. That is the case here. In our model, GSH has three effects: (i) reduction of arsenicals with valence 5 to valence 3; (ii) activation of AS3MT; (iii) sequestration of arsenicals by binding to GSH. We showed in the previous section (Figure 4) that with these three effects present, the model reproduces well the experimental data of [20] and [19] on the effect of the amount of GSH in the reaction mixture on MMAs, DMAs, and MMAs + DMAs concentrations (left, middle, and right panels in Figure 4). Figure 5 reproduces the right panel of Figure 4 and shows what would happen if the activation of AS3MT by GSH or the sequestration of arsenicals by GSH is eliminated. In our model, the excitation of AS3MT is given by *U*(*GSH*) (see Methods), a Hill function. If, instead, we make *U *(*GSH*) a constant equal to 5000, a number in the midrange of the values of *U*, then our computed model curve for MMAs + DMAs would be the black curve in the left panel of Figure 5. This curve, which is monotone decreasing because *U* does not increase with GSH and more and more of the arsenicals are sequestered by GSH, clearly does not fit the data. On the other hand, if we keep our usual model function *U* and remove the binding of GSH to arsenicals then the model produces the black curve in the right panel of Figure 5 for MMAs + DMAs. This curve, which is monotone increasing because the activation of AS3MT increases with GSH concentration and there is no sequestration effect, clearly does not fit the data. These results show conclusively that both effects, activation of AS3MT and sequestration of arsenicals by GSH are necessary to explain the experimental data. Similar results hold for the individual curves for MMAs and DMAs (simulations not shown).

### Temporal variation of GSH

In hepatocytes, GSH has concentrations in the mM range but is exported rapidly and turns over with a half-life of 1.5 to 2.5 hours [48]. The solutions in which cells are maintained typically contain the amino acids (cysteine, glycine, and glutamate or glutamine) necessary for the cells to resynthesize GSH. Nevertheless, the GSH concentration may vary considerably. For example, in the human hepatocytes used in [36], the cellular GSH concentration increased by 80% from day 1 to day 7. The experiments in [20] and [19] that we discussed above were conducted with purified enzymes in solution and not with living cells *in vitro*. The half-life of GSH in solution was found to vary from.2 to 70 hours depending on pH and temperature [49]. This raises the question of whether GSH degradation might play a role in experiments with purified enzyme in solution.

**Figure 6:**
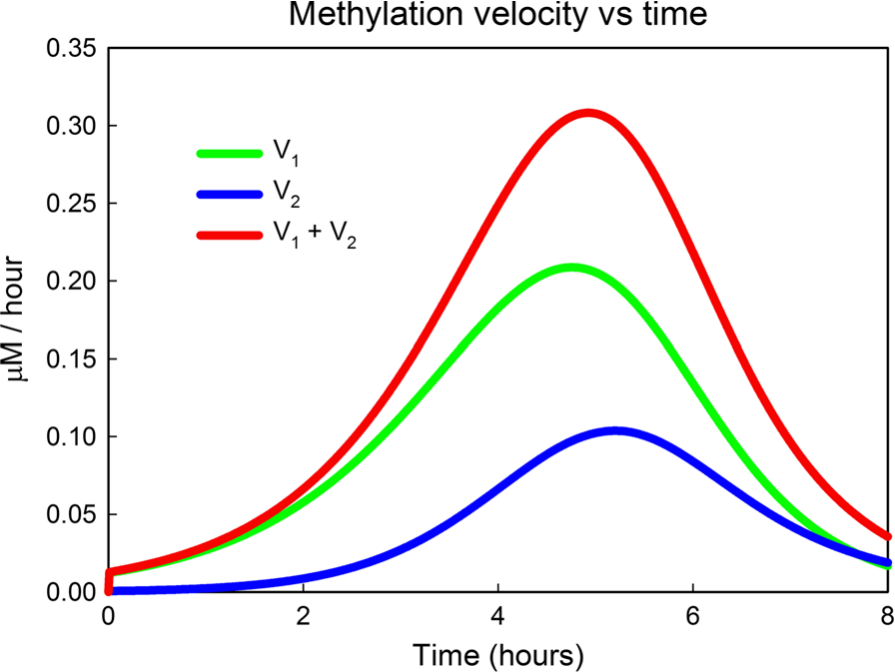
**The effect of the half-life of GSH. **The reactions mixture starts with 20 mM GSH and 1 *μ*M iAs. We assume the half-life of GSH in the mixture is 2.5 hours. The rates of the first methylation step, *V*_1_, the second methylation step, *V*_2_, and total methylation vary dramatically throughout an 8 hour period. For explanations, see the text.

We investigated the effect of GSH degradation by a model simulation in which the half-life of GSH was assumed to be 2.5 hours. The initial amount of GSH in the reaction mixture was 20 mM and the initial amount of iAs was 1 *μ*M. Figure 6 shows the time courses of the rate of first methylation step (*V*_1_), the rate of the second methylation step (*V*_2_), and the total rate of methylation as a function of time over 8 hours. In the beginning, methylation is very slow because there is so much GSH in the mixture and the GSH sequesters the arsenicals. Later, around 4–5 hours, there is much less sequestering and the activation of AS3MT causes methylation to proceed rapidly. Finally, at 7–8 hours, much of the GSH has degraded and so the methylation reaction runs slowly because of the lack of activation of AS3MT and the fact that many of the arsenicals have already been methylated. The main point is that, in both *in vitro* experiments and experiments in solution, the amount of GSH in the solution, the medium, and the cells should be tracked in time course experiments, because if it varies considerably that would affect the interpretation of the results.

## Discussion

The main point of this study was to explore the different ways that GSH could affect the Challenger pathway [6] for oxidative methylation of inorganic arsenic. Three effects were included: (i) reduction of arsenicals with valence 5 to valence 3; (ii) activation of AS3MT; (iii) sequestration of arsenicals by binding to GSH. We used the model to analyze the experimental data in [20] and [19]. First we showed that experiments with MMAs as a substrate in [19] show clearly that MMAs ^III^ is an inhibitor of the second methylation step. Next we showed that the model predictions, Figure 3, match well the experiments in [19] where the amounts of iAs, MMAs, and DMAs were followed over time. Both [20] and [19] show that methylation proceeds slowly at low GSH and high GSH, but quite quickly at intermediate GSH ranges. This important finding is reproduced by the model, Figure 4, and we show that the reason for this is the combined effect of AS3MT activation by GSH and the sequestration of arsenicals by GSH. Finally, we pointed out that temporal variation in the amount GSH in reaction mixtures or cells needs to be taken into account in interpreting experimental data.

An important consequence of these findings is that recent experimental data can be explained well by the Challenger pathway augmented with these effects of GSH. This does not prove that the Hayakawa pathway [21], in which only GSH-conjugated arsenicals are methylated, is wrong. It just shows that the methylation of GSH-conjugated arsenicals is not necessary to explain the effects of GSH seen in [20] and [19]. Indeed, it is possible that both GSH free arsenicals and GSH bound arsenicals can be methylated, perhaps at different rates. There is some evidence that for a methyl transferase that is orthologous to AS3MT that GSH-conjugated arsenicals are preferred substrates for binding to the enzyme’s active site [23].

Easterling et al. [38] created a pharmacokinetic model to study the hepatocyte data in [36]. In order to fit the data, they needed to introduce a storage compartment for arsenicals in cells. Likewise, in our whole body model and hepatocyte model [30] we needed to introduce cellular storage compartments. It is tempting to speculate that the binding of arsenicals to GSH was an important part of the“storage mechanism” in both cases.

S-adenosylmethionine (SAM) is the methyl group donor in the methylation reactions. It is not included explicitly in our model because SAM was not varied in the experiments that we were trying to explain. The SAM concentration occurs implicitly in the *V*_*max *_values of the first and second methylation reactions. The *K*_*m *_of AS3MT for SAM was measured as 11.8 *μ*M in [41], but the data in [20] imply that the *K*_*m *_is 50 *μ*M. This is an important issue for the applications of arsenic biochemistry to human toxicity studies. Gamble and coworkers [28,29] showed that folate supplementation of folate-deficient individuals in Bangladesh lowers blood arsenic levels. Raising folate levels can raise SAM concentrations in folate deficient individuals [50], so the presumed mechanism was that SAM levels were raised, thus making more methyl groups available for the methylation reactions. However, once SAM levels are back into the normal range (50 – 100 *μ*M for rats), raising SAM more by further folate supplementation won’t help if the *K*_*m *_= 11.8 because the reaction will already be saturated, whereas if the *K*_*m *_= 50 *μ*M then further supplementation should help.

The binding of GSH to arsenicals may be a significant detoxification mechanism as there is evidence that arsenic binds to GSH and then is removed in the bile [25,51] and sequestration might also reduce the toxicity of trivalent arsenicals. Thus, whole body models of arsenic detoxification need to take into account this removal mechanism as well as the removal of arsenic-GSH conjugates from the liver to the blood and urine. This will be the subject of future work.

The effects of GSH on arsenic methylation discussed in this study and the removal of arsenic-GSH complexes in the bile and urine imply that increasing GSH might be a way to reduce As toxicity. GSH levels are under strong regulatory control in the liver [52]. Nevertheless, supplementation strategies have proven useful in several circumstances where GSH liver levels are low. N-acetyl cysteine is the antidote given in emergency departments in cases of acetaminophen overdose [53,54] and glutamine is often given after surgery or other trauma to decrease inflammation [55,56]. In both cases the intent is to increase GSH production in the liver. Plasma GSH levels in Bangladesh are quite low, 2.6 *μ*M [57] as compared to the normal range, 2 – 20 *μ*M [47,58,59]. This suggests that supplementation by N-acetyl-cysteine may be a viable strategy for reducing arsenic toxicity.

## Conclusions

• The Challenger pathway, supplemented by three effects of glutathione, is sufficient to explain recent data on arsenic methylation.

• Monomethylarsonous acid inhibits the second methylation step.

• The three different effects of glutathione on arsenic methylation make the interpretation of experimental results difficult.

• Mathematical modeling of arsenic methylation can aid in the interpretation of experimental data.

• Supplementation by N-acetyl-cysteine may be a viable strategy for reducing arsenic toxicity.

## Competing interests

The authors declare that they have no competing interest.

## Authors’ contributions

MG and MH initiated the project and gave advice on the epidemiology and biochemistry of arsenic. FN directed the project. JY and SL developed the mathematical model and wrote the code with the advice of MR and FN. MR wrote the manuscript with the advice of FN, MG, MH, and SL. All authors read and approved the final manuscript.
